# Identification of Head and Neck Cancer Subtypes Based on Human Papillomavirus Presence and E2F-Regulated Gene Expression

**DOI:** 10.1128/mSphere.00580-17

**Published:** 2018-01-10

**Authors:** Molly E. Johnson, Paul G. Cantalupo, James M. Pipas

**Affiliations:** a Department of Biological Sciences, University of Pittsburgh, Pittsburgh, Pennsylvania, USA; Northwestern University

**Keywords:** cancer, HPV, metagenomics, tumor suppressor genes

## Abstract

Human papillomavirus (HPV) is present in a subset of head and neck squamous cell carcinomas (HNSCCs). The cell cycle regulatory Rb-E2F pathway is a major target of HPV and is perturbed by these viruses in cell culture and animal models, as well as in human tumors. In this study, we examined differences in the Rb-E2F pathway displayed by HPV-positive (HPV^+^) and HPV-negative (HPV^−^) HNSCC tumors. We created a computational approach that effectively categorizes gene expression as unchanged, downregulated, or upregulated by comparing the gene’s mRNA levels in the tumor to the corresponding mRNA levels across normal tissue samples. Our findings suggest that there are three major HNSCC subtypes, defined by differences in the presence of HPV and in E2F-regulated gene expression. Most HPV^+^ HNSCC tumors show upregulation of E2F-regulated genes, which is consistent with inactivation of Rb by the virus-encoded E7 protein. In contrast, many HPV^−^ HNSCCs show little or no change in the Rb-E2F pathway. However, we also identified a set of tumors that show alterations in the Rb-E2F pathway in the absence of HPV. Thus, one class of HPV^−^ HNSCCs arise without significant alterations of the Rb-E2F pathway, while a second class of tumors appear to deregulate this pathway independently of the presence of HPV.

**IMPORTANCE** Cancer is a complex disease that can be caused by a multitude of factors. HNSCC is complicated because some of these cancers are clearly associated with HPV, while others have no viral involvement. Determining the pathways that are commonly altered in both types of HNSCC, as well as those that are unique to viral and nonviral tumors, is important for a basic understanding of how these cancers arise and progress and critical to the development of targeted therapies. In this work, we show that all HPV-associated tumors have increased expression of E2F target genes, indicating that the tumor suppressor function of Rb is blocked. Importantly, Rb is also inhibited in a subset of nonviral tumors, suggesting that mutations present in these cancers mimic the action of the HPV E6 and E7 oncogenes.

## Abstract (precis)

Cancer is a complex disease that can be caused by a multitude of factors. HNSCC is complicated because some of these cancers are clearly associated with HPV, while others have no viral involvement. Determining the pathways that are commonly altered in both types of HNSCC, as well as those that are unique to viral and nonviral tumors, is important for a basic understanding of how these cancers arise and progress and critical to the development of targeted therapies. In this work, we show that all HPV-associated tumors have increased expression of E2F target genes, indicating that the tumor suppressor function of Rb is blocked. Importantly, Rb is also inhibited in a subset of nonviral tumors, suggesting that mutations present in these cancers mimic the action of the HPV E6 and E7 oncogenes.

## INTRODUCTION

Head and neck squamous cell carcinoma (HNSCC) is the sixth most common cancer worldwide and is associated with exposure to various risk factors, such as tobacco or human papillomavirus (HPV) infection (1). In recent years, an increase in tumors located in the tonsil and the base of the tongue has been seen due to infection with HPV (1). Multiple subtypes of HNSCCs have been characterized by various genotypic traits, such as alterations in cell growth pathways, and clinical traits, such as the diverse locations of tumors (2, 3). This emphasizes the need for the development of targeted therapies, particularly as treatment for HNSCC can be toxic and extremely difficult, with 50% relapse within 2 years of tumor removal (1). Recently, The Cancer Genome Atlas (TCGA) published a comprehensive genomewide characterization of 279 HNSCC patients, identifying genetic/clinical subtypes of the cancer based on the functionality of numerous pathways (3). Specifically, they defined subtypes of HPV-positive (HPV^+^) and HPV-negative (HPV^−^) tumors, primarily based on unique mutations and copy number variants, and analyzed how these correlated with various pathways. These studies concluded that the presence of HPV represents a distinct subgroup of the cancer with its own unique genetic signatures, specifically through its modification of the cell cycle regulatory pathway.

The Rb-E2F pathway, as a critical regulatory system for cell cycle progression, is a major target of HPV-mediated tumorigenesis (4). In normal cells, Rb proteins inhibit the transcription factor E2F, preventing E2F from upregulating a collection of genes, i.e., the E2F-responsive genes (ERGs), that are needed for cell proliferation (5). The proteins encoded by many ERGs are involved in nucleotide synthesis, DNA replication, and cell cycle progression. In some cancers, this pathway is altered so that E2F-dependent transcription occurs in an unregulated fashion (6). The HPV E7 protein specifically binds to and degrades Rb, allowing E2F-dependent transcription to occur and the cell cycle to proceed unchecked (7–9). However, other factors may regulate the Rb-E2F pathway. For instance, growth signals induce the formation of cyclin/cyclin-dependent kinase (CDK) complexes, which inactivate Rb through phosphorylation, and growth-inhibiting signals promote the expression of cyclin kinase inhibitors (CKIs), which interact with cyclin/CDK complexes and prevent them from phosphorylating Rb, thus inhibiting ERG expression (6, 10). In normal cells, unregulated cell growth frequently results in apoptosis, often as a result of the stabilization and activation of the tumor suppressor p53 (11). HPV blocks this response through the E6 protein that binds p53 and stimulates its degradation (12–14).

Much of our knowledge of how HPV affects the Rb-E2F and p53 (Rb-E2F/p53) pathways is based on cell culture and animal models. The TCGA data provide an opportunity to explore the role of these pathways in the context of human tumors. Thus far, most research on the TCGA data has focused on genomewide analyses. In this study, we focus specifically on the Rb-E2F/p53 pathways to identify HNSCC subtypes.

## RESULTS

### Rb-E2F/p53 pathway expression patterns distinguish HPV^+^ and HPV^−^ tumors.

**FIG 1:**
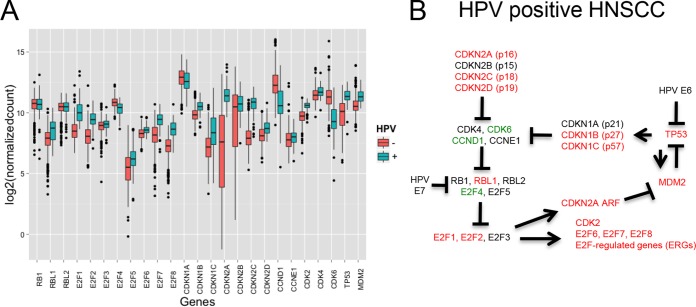
Effect of HPV on the expression of Rb-E2F/p53 pathway genes in HNSCCs. (A) Log expression levels of the 25 genes in the Rb-E2F/p53 pathways (see Table S1 in Data Set S1 in the supplemental material) are represented by boxplots. Data for HPV^+^ samples are colored in teal, and data for HPV^−^ samples are in red. (B) The Rb-E2F/p53 pathways in HPV^+^ tumors. The CDKN2A gene produces differentially spliced transcripts for the p16 and ARF proteins. Red, upregulated; green, downregulated; black, no significant difference.

Twenty-five genes (see Table S1 in Data Set S1 in the supplemental material) representing members of key interacting groups in the Rb-E2F/p53 pathways (cyclins/CDKS, CKIs, E2Fs, RBs, and TP53), as well as genes connecting the Rb-E2F and p53 pathways (CDKN2A/p14ARF and MDM2), were selected for analysis. We captured TCGA transcriptome sequencing (RNA-seq) expression values for 499 tumor and 43 normal tissue samples and visualized the cellular mRNA levels across HPV^+^ and HPV^−^ tumor samples with boxplots (Fig. 1A). Using a *P* value cutoff of 1e−4, we found that the mRNA levels of 17/25 genes were significantly different between HPV^+^ and virus-negative samples (Table S1 in Data Set S1). Genes exhibiting significantly higher mRNA levels in HPV^+^ tumors included many known targets of Rb-mediated transcriptional repression, such as E2F1, E2F2, and RBL2 (p107). Interestingly, while E2F1 to -3 are all known as activators of cell cycle progression (5), the levels of E2F3 were not significantly different between virus-associated and virus-free tumors.

10.1128/mSphere.00580-17.4 DATA SET S1  Tables S1 to S10. Download DATA SET S1, PDF file, 0.9 MB. Copyright © 2018 Johnson et al. 2018 Johnson et al. This content is distributed under the terms of the Creative Commons Attribution 4.0 International license.

These results suggest that HPV-associated tumors can be distinguished from nonviral tumors based on changes in the expression of genes in the Rb-E2F/p53 pathways. To test this, we performed consensus clustering based on categorized RNA levels of genes (see Materials and Methods) in these pathways (Fig. S1). This analysis showed that HPV^+^ tumors cluster as a single group.

10.1128/mSphere.00580-17.2 FIG S1  Consensus clustering identifies HPV^+^ tumors as a unique group based on RNA expression levels in the Rb-E2F/p53 pathway. Gene expression in each tumor was categorized (see Materials and Methods in the text), and the 25 genes in the Rb-E2F/p53 pathway (see Table S1 in Data Set S1 in the supplemental material) were clustered based on the categorized RNA levels using the pheatmap R package. Each gene in every tumor is defined as upregulated (red), unchanged (yellow), or downregulated (blue). Separately, consensus clustering was applied to the Rb-E2F/p53 categorized gene expression data using the ConsensusClusterPlus R package. The hierarchical clustering algorithm was used with Euclidian distance, and the parameters were set to 80% item resampling, 100% gene resampling, a maximum evaluation of 10 clusters, and 500 subsamples. The results identified 6 groupings of the tumors to be the most significant clustering level of the data. These tumor groupings were added to the heatmap as an annotation and show that the HPV^+^ tumors are classified as a significantly unique cluster as verified by the consensus clustering algorithm. Download FIG S1, PDF file, 0.1 MB. Copyright © 2018 Johnson et al. 2018 Johnson et al. This content is distributed under the terms of the Creative Commons Attribution 4.0 International license.

### HPV^+^ tumors and a subset of HPV^−^ tumors have altered E2F-regulated gene expression.

The combination of increased levels of E2F1 and E2F2 with HPV-mediated degradation of Rb should lead to increased expression of an array of genes whose expression is dependent on E2F. Therefore, we analyzed the RNA levels of ERGs in HNSCC tumors. We used a specific list of 325 ERGs (Table S2 in Data Set S1) (15) and categorized their mRNA levels in each tumor sample as upregulated, downregulated, or unchanged (see Materials and Methods). Then, for each tumor sample, we calculated the total number of upregulated ERGs.

**FIG 2:**
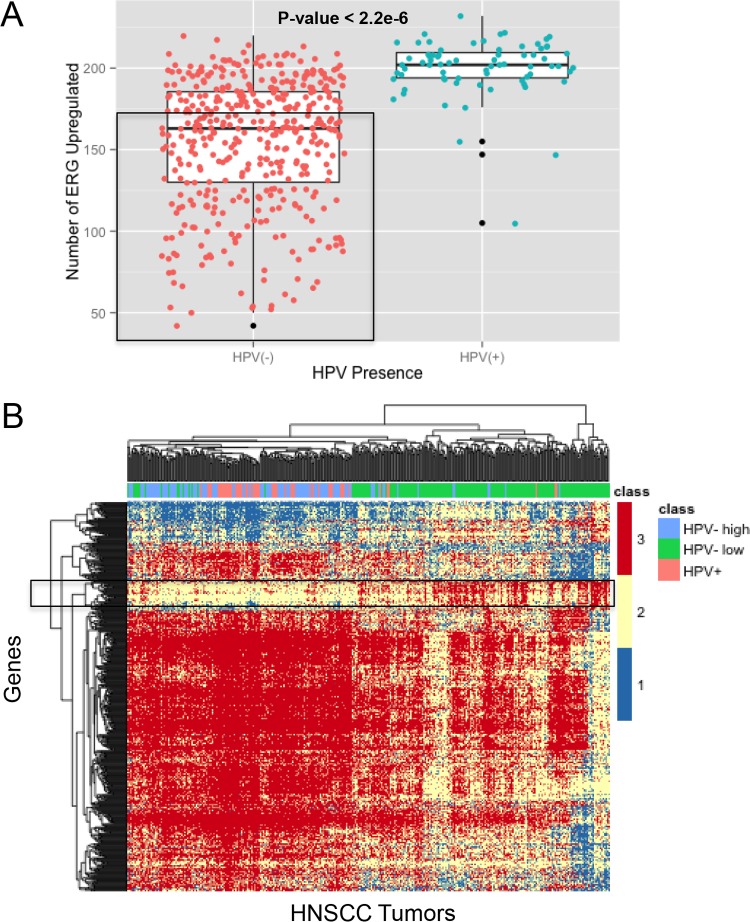
HNSCC tumors partition into three groups based on E2F-regulated gene expression. (A) Gene expression in HNSCC tumors was categorized as described in Materials and Methods. The total number of genes upregulated out of the 325 E2F-regulated genes (Table S2 in Data Set S1) was quantified for each patient. HPV^−^ patient data are marked by red circles, HPV^+^ patient data by blue circles, and outliers by black circles. Three groups are defined based on the number of ERGs upregulated and the presence of HPV: HPV^+^, high-ERG tumors; HPV^−^, high-ERG tumors; and HPV^−^, low-ERG tumors (the latter group is bounded by the rectangle). The *t* test was used to compare the difference between the results for HPV^+^ and HPV^−^ samples. (B) Analysis of ERG-defined HNSCC groups shows a subset of genes uniquely upregulated in the HPV^−^, low-ERG group. The global mRNA levels in the three HNSCC groups were analyzed. Gene expression was categorized with a cutoff of 1 (see Materials and Methods), and an ANOVA/Tukey test was run to find genes differentially regulated between the HPV^−^, low-ERG group and HPV^−^ or HPV^+^, high-ERG tumors. The resulting 586 genes were clustered, and the results displayed as a heatmap. Genes that are uniquely upregulated in HPV^−^, low-ERG tumors are highlighted with a rectangle.

In HPV^+^ HNSCC samples, there was a range of 175 to 230 (median, 202) ERGs upregulated in each tumor, with three low outliers (Fig. 2A). The high number of ERGs upregulated in HPV^+^ tumors supports the results presented in Fig. 1 and suggests that HPV affects the Rb-E2F pathway. In HPV^−^ HNSCC samples, there was a much larger range of 40 to 230 (median, 163) ERGs upregulated in each tumor. The two tumor groups were significantly different from each other (*t* test, *P* < 2.2e−16). The range in ERG upregulation suggests that three groups of tumors can be defined based on ERG upregulation: HPV^+^, high-ERG tumors (more than 175 ERGs upregulated), HPV^−^, high-ERG tumors (more than 175 ERGs upregulated), and HPV^−^, low-ERG tumors (less than 175 ERGs upregulated).

### Analysis of tumor subgroups.

Since HPV^−^, high-ERG tumors and HPV^+^ tumors seem to be affecting the Rb-E2F pathway in similar ways, we wanted to learn which pathways the HPV^−^, low-ERG group may be uniquely affecting. We searched for genes regulated exclusively in the HPV^−^, low-ERG subset of tumors. We used an expression cutoff of 1 to remove genes that had negligible RNA levels in both normal tissue and tumors (see Materials and Methods). We then performed analysis of variance (ANOVA) on the categorized RNA levels of the remaining genes that had measurable expression levels, searching for significant differences in RNA levels between the three ERG tumor classes: HPV^−^, low ERG; HPV^−^, high ERG; and HPV^+^, high ERG. ANOVA identified 2,513 genes with significantly different mRNA levels (*P* < 1e−10) between the three groups. The Tukey test was applied to the ANOVA results to calculate the differences between pairs of groups for each gene. Out of these 2,513 genes, 586 genes (Table S3 in Data Set S1) were identified as having significantly different RNA levels in the HPV^−^, low-ERG tumors than in the HPV^+^ and HPV^−^, high-ERG tumors (*P* < 0.01) but similar RNA levels in HPV^+^ and HPV^−^, high-ERG tumors (*P* > 0.01).

These 586 genes were grouped by clustering, and the results are displayed as a heatmap in Fig. 2B. The HPV^+^ and HPV^−^, high-ERG tumors clustered together and apart from the HPV^−^, low-ERG tumors. For the HPV^+^ tumors, the different HPV species that we detected did not cluster together (data not shown). In the HPV^−^, low-ERG tumors, a group of 45 genes with unique upregulation was identified (Table S4 in Data Set S1). A subsequent analysis further supported the identification of this group, where these genes were in the top 58 genes with the highest percentages of upregulation in the low-ERG group and smallest percentages of upregulation in the high-ERG group. Subsequent annotations of these genes using DAVID (16) revealed a weak association with cell processes, such as ectoderm development (*P* = 9.0e−6) and keratin (*P* = 4.3e−2), cytoskeleton (*P* = 5.4e−2), and protein complex (*P* = 1.3e−1) assembly. Genes uniquely upregulated in the HPV^−^, low-ERG tumors were also analyzed with Ingenuity Pathway Analysis software (see Materials and Methods), which revealed two major networks: dermatological diseases/inflammatory response and cell death/survival/development (Table S5 in Data Set S1). We were unable to identify transcription factor signatures in these genes using ENRICHR.

The ERG-defined groups were further analyzed by clinical phenotypes (Table S6 in Data Set S1). Both HPV^−^, high-ERG and HPV^+^ tumors were more associated with higher tumor grades and clinical N grades (lymph nodes) than the HPV^−^, low-ERG group. Previously identified HPV^+^ HNSCC clinical associations were also verified, such as a high presence in the base of the tongue and the tonsil, a better vital status, and a higher occurrence in males (1). We did not find clinical phenotypes significantly correlated with the HPV^−^, low-ERG group. The ERG-defined groups were also analyzed for mutational signatures, but no significant patterns were found due to the small mutational overlap across patients (Text S1 and Tables S7 and S8 in Data Set S1).

10.1128/mSphere.00580-17.1 TEXT S1  Mutational signature analysis, analysis of categorization method, and cutoff determination for categorization. Download TEXT S1, PDF file, 0.1 MB. Copyright © 2018 Johnson et al. 2018 Johnson et al. This content is distributed under the terms of the Creative Commons Attribution 4.0 International license.

### HPV^+^ tumors have lower levels of pRb and higher levels of p16 protein than HPV^−^ tumors.

**FIG 3:**
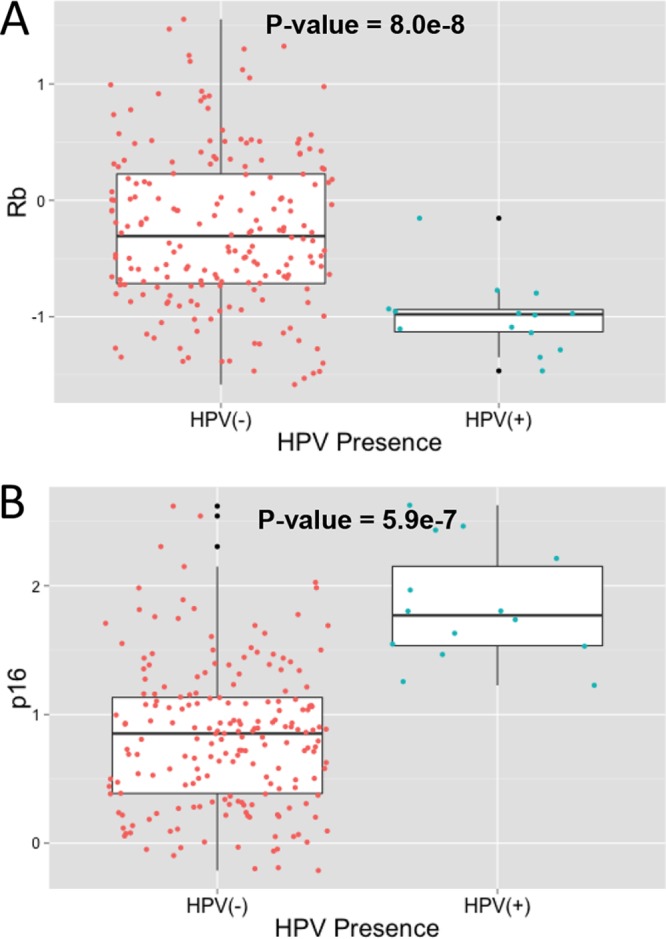
Variations in protein expression of Rb and p16 based on HPV presence. Levels of protein expression of Rb (A) and p16 (B) in HNSCC tumor samples were retrieved from TCGA as described in Materials and Methods. Expression levels were compared across tumors (*z* score) and visualized as boxplots based on the presence of HPV. The difference in protein expression between HPV^+^ and HPV^−^ samples was compared using a *t* test. Data for HPV^−^ samples are in red, data for HPV^+^ samples are in blue, and data for outliers are in black.

Next, we examined the levels of the phosphorylated pRb and p16 proteins in HNSCC tumors using normalized reverse-phase protein array (RPPA) data from TCGA. Due to HPV E7’s ability to cause degradation of Rb, we expected to see low Rb levels in HPV^+^ tumors, and we found that phosphorylated pRb is present at very low levels in HPV^+^ tumors compared to the levels in HPV^−^ tumors (*t* test, *P* = 8.0e−8) (Fig. 3A). We were unable to determine the levels of total or hypophosphorylated pRb, since RPPA data were unavailable. The protein expression of the cyclin-dependent kinase inhibitor p16 (CDKN2A gene) was also analyzed to determine whether the frequent upregulation seen in HPV^+^ tumors at the RNA level (*P* = 2.8e−18) (Table 1) translates into increased protein levels. We found that p16 protein expression is higher in HPV^+^ tumors than in HPV^−^ tumors (*t* test, *P* = 5.9e−7) (Fig. 3B). Previous studies have suggested an epigenetic explanation for the p16 addiction seen in HPV^+^ tumors, specifically by the demethylase KDM6 (17–19). We analyzed CDKN2A upregulation in relation to the RNA levels of KDM6-regulated genes, as well as the levels of KDM6 demethylase itself (Fig. S2). No significant associations were found between HPV^+^ samples and the presence of KDM6, suggesting that a different mechanism may be responsible for the HPV^+^ tumors’ unique CDKN2A upregulation. We also analyzed p53 protein levels to determine whether p53 is degraded in HPV^+^ tumors. However, we observed that the p53 protein levels in HPV^+^ tumors were similar to the median protein levels of p53 in HPV^−^ tumors (data not shown).

10.1128/mSphere.00580-17.3 FIG S2  Clustering of HOX/KDM6 genes shows insignificant association with HPV presence and CDKN2A upregulation. HOX genes were analyzed as they are known to be regulated by the KDM6 demethylase. (A) HOX gene expression and (B) CDKN2A, KDM6A, and KDM6B gene expression was categorized as described in Materials and Methods. The categorized gene expression data were clustered on the *y* axis, and patient sample data were clustered on the *x* axis, using the pheatmap R package. Each gene in every tumor is defined as upregulated (red), unchanged (yellow), or downregulated (blue). HPV presence is added as an annotation. Download FIG S2, PDF file, 0.1 MB. Copyright © 2018 Johnson et al. 2018 Johnson et al. This content is distributed under the terms of the Creative Commons Attribution 4.0 International license.

**TABLE 1:** CDKN2A expression contingency table

Presence of HPV^a^	No. of tumors in which CDKN2A expression was^b^:		
	Downregulated	Normal	Upregulated
−	24	185	218
+	0	0	72

a HPV was considered present if the number of alignments to virus was ≥1,000.

b CDKN2A expression levels were categorized using the categorization method (see Materials and Methods).

### Mutational profiles are consistent with active HPV E7 and E6 gene functions.

E7 inactivates Rb function by binding it and stimulating its degradation. Accordingly, the presence of the HPV E7 protein and upregulated ERGs in HPV^+^ tumors indicates that Rb-mediated transcriptional repression is impaired in these samples. Furthermore, the HPV E6 protein present in these tumors should block p53 function. This is consistent with the gene expression profiles described above.

Therefore, we hypothesized that there would be no selective pressure to mutate *RB1*, *CDKN2A*, or *TP53* in HPV^+^ HNSCC tumors. To test this, mutation data from TCGA were analyzed for each tumor. If a gene had one or more nonsynonymous mutations, it was scored as mutated. Otherwise, it was considered wild type. All genes in the Rb-E2F pathway were analyzed (Table S9 in Data Set S1), but only CDKN2A, TP53, and RB1 had significantly different mutational profiles (*P* < 0.05). No mutations were detected in *CDKN2A* in the presence of HPV (0/68), but *CDKN2A* mutations were found frequently in HPV^−^ HNSCC tumors (113/440, *P* = 1.8e−8) (Table 2). While these mutations are based on the p16INK4a transcript, we also found that 70 of the 113 patients with *CDKN2A* mutations also have an altered p14ARF transcript. Similarly, only one HPV^+^ tumor (1/68) had a *TP53* mutation, whereas the majority of HPV^−^ tumors (359/440) had a *TP53* mutation (*P* = 1.2e−40) (Table 2).

**TABLE 2:** Mutation contingency table

Gene	Presence of HPV^a^	No. of tumors in which gene was^b^:	
		Not mutated	Mutated
CDKN2A	−	327	113
CDKN2A	+	68	0
TP53	−	81	359
TP53	+	67	1
RB1	−	428	12
RB1	+	62	6

a HPV was considered present if the number of alignments to virus was ≥1,000.

b Mutation was defined as one or more nonsilent mutations found in that gene.

**FIG 4:**
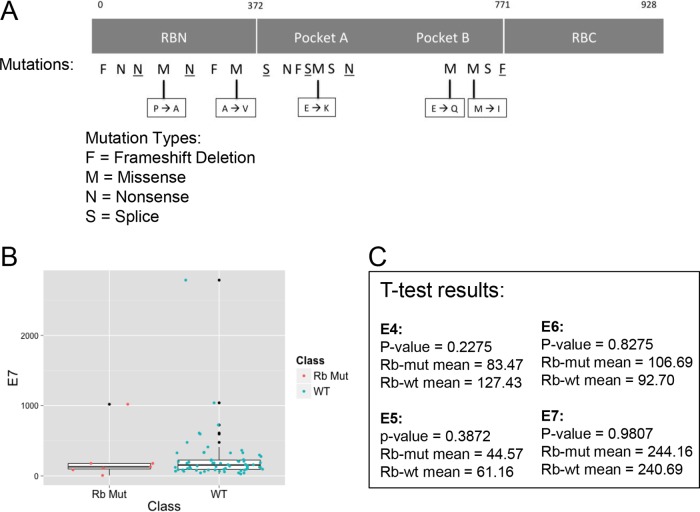
Analysis of RB1 mutations shows detrimental effects independent of HPV protein expression. (A) Locations and types of RB1 mutations are annotated on the full-length Rb protein to determine functional effects. Mutations at specific locations are indicated by a letter representing the type of mutation. Underlined letters represent an HPV^+^ sample, and plain letters represent an HPV^−^ sample. Amino acid changes are identified for missense mutations. RBN, Rb amino-terminal domain; RBC, Rb carboxy-terminal domain. (B) RNA levels of HPV E7 in HPV^+^ RB1 mutant (red) and RB1 wild-type (blue) HNSCC tumors were analyzed using a boxplot. Data for outliers are in black. (C) The *t* test was used to compare the average RNA levels for other HPV proteins (E4, E5, E6, and E7) between the two groups (HPV^+^ RB1 mutant and RB1 wild-type HNSCC tumors), to check for any significant differences between these two groups.

Interestingly, *RB1* appears to be mutated in almost 10% of the HPV^+^ tumors (6/68) and in a few of the HPV^−^ tumors (12/440, *P* = 0.02) (Table 2). The higher *RB1* mutation frequency seen in HPV^+^ tumors compared to the frequency in HPV^−^ tumors is unanticipated, due to HPV’s ability to degrade pRb. We hypothesized that the mutations may be nonfunctional, occurring in the presence of HPV solely due to random chance, and thus, we investigated whether the mutations in *RB1* had a functional effect. However, many of the mutations were frameshift deletions or missense mutations, significantly changing the amino acid characteristics in important regions (Fig. 4A). Thus, it appears that all of the mutations present in HPV^+^ tumors would inactivate pRb function. Next, we analyzed the LXCXE motif, the HPV E7 region that binds to pRb, in these tumor samples. We found that the E7 gene was wild type in all samples (data not shown). Finally, we explored the idea that E7 may not be highly expressed in tumor samples harboring *RB1* mutations. We compared E7 RNA levels between HPV^+^, *RB1* mutant samples and HPV^+^, *RB1* wild-type samples. The results show similar levels in both types of HPV^+^ tumors: the *RB1* mutant, HPV^+^ tumors have an E7 mean value of 244.2, while the *RB1* wild-type, HPV^+^ tumors have an E7 mean value of 240.7 (*t* test, *P* = 0.98) (Fig. 4B and C). Similarly, there were no significant differences in the RNA levels of the other HPV proteins in the two types of HPV^+^ tumors (Fig. 4C).

## DISCUSSION

We have examined the effects of HPV on the Rb-E2F/p53 axis in human squamous cell carcinomas of the head and neck. We captured DNA sequencing, RNA-Seq, mutation, and protein data representing 499 patients from TCGA. HPV^+^ tumors were identified by aligning DNA and/or RNA-seq reads to sequences in HPV databases. Consistent with previous reports, we found that 14.2% of HNSCC tumors contained HPV (3, 20). The RNA-Seq data indicate that all HPV-positive tumors express the HPV E7 and E6 oncogenes. Thus, the Rb and p53 tumor suppressors should be inactive in these tumors. To test this, we compared the mRNA levels and, in some cases, protein levels of components of the Rb-E2F/p53 pathways and their downstream targets in HPV-associated tumors with the levels in tumors that did not harbor HPV, as well as the levels in normal tissue.

We examined several key components of the Rb-E2F/p53 pathways in HPV^+^ and HPV^−^ tumors. Tumors containing HPV consistently had lower levels of phosphorylated pRb protein than virus-negative tumors. This is consistent with the action of the HPV-encoded E7 protein, which is known to stimulate pRb degradation.

Surprisingly, there was no significant difference in p53 protein levels between virus-associated and virus-negative tumors, although p53 mRNA levels were elevated in the virus-positive group. It is unclear if this indicates that the HPV E6 protein does not degrade p53 in these tumors or if p53 protein levels are universally low in both HPV^+^ and HPV^−^ HNSCCs. The latter interpretation is consistent with data reviewed below that strongly indicate that p53 is inactive in nearly all HNSCCs (3). Since the p53 antibody used came from Cell Signaling Technology, Inc. (catalog number 9282), and was used for all TCGA RPPA assays (direct communication with the RPPA core), we believe these results are not due to the failure of the antibody to detect both mutant and wild-type forms of p53. Finally, consistent with a number of reports, the protein levels of p16 were consistently elevated in HPV-associated tumors (19, 21, 22).

Gene expression profiling analysis also indicated that the pRb and p53 tumor suppressors were inactive in HPV-associated HNSCCs. First, the mRNAs encoding several cyclin-dependent kinase inhibitors, including members of both the INK4a (p16, p18, and p19) and CIP1/WAF1 (p27 and p57) families, were elevated in HPV^+^ tumors. These proteins share the ability to antagonize the activity of the cyclin-dependent kinases responsible for pRb inactivation (6). Furthermore, the levels of cdk6 and cyclin D1 mRNAs were lower in HPV^+^ tumors. These observations suggest that pRb should be active in HPV-associated HNSCCs. However, the presence of the HPV E7 protein leads to pRb degradation, thereby bypassing these brakes to cell proliferation. Consistent with this interpretation, HPV^+^ tumors also contained elevated levels of mRNAs encoding members of the E2F transcription family and of a number of genes whose expression is known to be E2F dependent (Fig. 1 and 2A).

If the pRb and p53 tumor suppressors are inactivated by HPV, there should be little or no selective advantage for these genes to be mutated during the course of tumorigenesis. Similarly, there would be no selective advantage to mutate p16, since HPV E7 has eliminated its downstream target, pRb. In fact, an analysis by the TCGA consortium reported that while the vast majority of nonviral HNSCCs have mutations in p53, HPV-associated tumors do not (3). We confirmed this observation and extended it to include p16. Similar to p53, we found that p16 was frequently mutated in nonviral HNSCCs, while none of the HPV-associated tumors carried mutations in this gene. Surprisingly, we found that *RB1* was mutated in about 10% of HPV^+^ tumors and in 2.7% of HPV^−^ tumors. In these cases, the nature of the mutations suggests that they would inactivate *RB1* function. Examination of the HPV E7 proteins in these cases indicated that they were wild type with an intact LXCXE motif, indicating they should be fully capable of inactivating pRb. One interesting possibility is that the mutations of *RB1* occurred before HPV infection and integration. In such cases, the RB family members p130 and p107 might compensate for the loss of pRb. The subsequent appearance of E7 would then add an additional selective advantage by eliminating p130 and p107. Alternatively, the E7-mediated inactivation of pRb in these tumors may be inefficient for some unknown reason, thus providing a selective pressure for mutation. While early studies showed no evidence of mutations in these pathways, interestingly, pRb mutations were found in HPV^+^ cervical carcinomas in a recent study (23–25).

Rather than defining tumors through a global analysis of gene expression, this study took a targeted approach by comparing HNSCC samples based on the presence or absence of HPV and focused specifically on gene expression and mutational patterns in the Rb-E2F/p53 pathways, which are disrupted by the E7 and E6 oncoproteins from HPV. We demonstrated that HPV^+^ HNSCCs form a distinct group when clustered by Rb-E2F/p53 component analysis and that the properties of this group of tumors largely followed the current paradigms for HPV action on this pathway. Interestingly, this study revealed two distinct types of HPV^−^ HNSCCs based on the expression of E2F-regulated genes (Table 3). One group showed elevated levels of ERGs similar to those seen in HPV^+^ tumors. The second group showed little or no alteration of ERG expression. This is consistent with previous reports that indicate normal cell proliferation and tumorigenesis can occur in the absence of activator E2Fs under some circumstances (26–28). An understanding of the different patterns of expression in these subsets will move us closer to learning the mechanisms by which cancer progresses in each HNSCC subtype.

**TABLE 3:** ERG-based HNSCC subgroup summary

	Description for tumors that were:		
	HPV^−^ with:		HPV^+^
Parameter	Low ERG expression	High ERG expression	
ERG RNA levels	Low number of ERGs upregulated	High number of ERGs upregulated; distributionsimilar to that in HPV^+^subgroup	High number of ERGs upregulated
Clinical associations	NA^a^	Correlated with higher tumor grades and lymph node involvement	Correlated with higher tumor grades and lymph node involvement; highly present in base of tongue and tonsil, better vital status, more associated with males
Genomewide pathway analysis	Uniquely upregulated genes associated with ectoderm development and keratin, cytoskeleton, and protein complex assembly	NA	NA

a NA, not applicable.

## MATERIALS AND METHODS

### Data sets.

Transcriptome (RNA-SeqV2 version 3.1.8 normalized counts) and mutation data (version level 2.1.4.0) for 499 tumor and 43 normal tissue samples from HNSCC patients were downloaded from the TCGA data portal (https://tcga-data.nci.nih.gov). However, this website is no longer functional; thus, we are keeping a local copy of the data for posterity. Protein data (level 3, version 2016.01.28) were downloaded through Firebrowse (http://firebrowse.org/?cohort=HNSC) at the following URL: http://gdac.broadinstitute.org/runs/stddata__2016_01_28/data/HNSC/20160128/gdac.broadinstitute.org_HNSC.Merge_protein_exp__mda_rppa_core__mdanderson_org__Level_3__protein_normalization__data.Level_3.2016012800.0.0.tar.gz.

### Categorizing the expression of genes.

Genes were categorized as being as upregulated, downregulated, or unchanged in tumors relative to their expression in normal tissue. For each gene, both the median expression and the median absolute deviation (MAD) of the 43 normal samples were calculated. Median was chosen instead of mean because outlier values could skew the mean significantly. Then, the tumor expression of each gene in 499 HNSCC patients was compared to the median of its expression in normal tissue and categorized accordingly: upregulated if the tumor expression value for that gene is greater than the gene’s normal median plus 1 MAD, downregulated if the tumor expression value for that gene is less than the gene’s normal median minus 1 MAD, and normal if the tumor expression value of the gene falls anywhere within 1 MAD of the gene’s normal median. We found this categorization method to be more effective than the TCGA expression values in predicting HNSCC subtypes (see Text S1 and Table S10 in Data Set S1 in the supplemental material). In certain situations, a cutoff for this categorization was applied, in which case genes with RNA levels below the cutoff in both normal tissue and tumors were not considered in the analysis (Text S1).

### Clustering gene expression.

Gene expression patterns were clustered using the R pheatmap package with the default clustering distances. The default Euclidian distance clustered HPV^+^ tumors together better than other clustering distance methods. The categorized data (described above) were clustered using specific genes of interest. Data for genes with similar patterns of expression across tumor samples were clustered on the *y* axis, and data for patient tumor samples with similar patterns of expression across the genes of interest were clustered on the *x* axis. HPV presence was detected in TCGA BAM files as described previously (29). A virus was considered to have been detected in a sample if the number of alignments to the virus was ≥1,000.

### Protein and mutation data.

Protein expression in tumors was measured as a *z* score compared against the expression in other HNSCC tumors. The Rb protein measured was phosphorylated (S807/S811) Rb. A gene was considered mutated if one or more nonsynonymous mutations were found in that gene. No mutation was defined by zero nonsilent mutations or only silent mutations.

### Correlations.

To verify the association of clusters seen in the heatmap representation, correlations were done using the chi-square test or the Fisher test in R. Specifically, this tested for significant association of the genes’ mRNA levels (downregulated, upregulated, or normal) with the presence of HPV or mutations (*P* < 0.01). The Fisher exact test was also used for the mutation contingency analysis.

### Functional analysis.

To gain a better understanding of the biological function and significance of groups of genes, the Database for Annotation, Visualization, and Integrated Discovery (DAVID) was utilized (16). Genes were entered into the Functional Annotation Tool and analyzed with Functional Annotation Clustering, which gives enrichment scores and *P* values for the associated functions of the genes. Similarly, Ingenuity Pathway Analysis software (Qiagen) was used to identify pathways and diseases that are associated with a group of genes.
