# Supplementary material for: Identification of Head and Neck Cancer Subtypes Based on Human Papillomavirus Presence and E2F-Regulated Gene Expression
Source: mSphere. 2018 Jan 10;3(1):e00580-17. doi: 10.1128/mSphere.00580-17 (PMC5760753; doi:10.1128/mSphere.00580-17)
Supplement: DATA SET S1 [file sph001182443sd1.pdf]

Table S1. Rb-E2F pathway genes

Gene	Common Name	P-value (t-test)	HPV(-) mean	HPV(+) mean
RB1	Rb	6.17E-01	1762.2	1837.3
RBL1	p107	2.73E-07	261.5	446.6
RBL2	p130	9.95E-01	1461.5	1461.0
E2F1	E2F1	6.86E-07	443.9	1482.9
E2F2	E2F2	5.16E-17	313.5	791.4
E2F3	E2F3	8.25E-02	516.9	561.9
E2F4	E2F4	9.44E-14	1932.0	1383.3
E2F5	E2F5	9.93E-04	63.5	99.6
E2F6	E2F6	1.13E-06	325.8	396.8
E2F7	E2F7	3.33E-15	341.6	738.7
E2F8	E2F8	8.72E-15	171.6	440.3
CDKN1A	p21	3.27E-02	8562.7	7346.5
CDKN1B	p27	2.74E-11	997.3	1561.9
CDKN1C	p57	1.55E-05	202.7	585.1
CDKN2A	p16INK4a/p14Arf	8.18E-14	645.7	3520.2
CDKN2B	p15	6.13E-01	2021.2	1931.8
CDKN2C	p18	3.19E-22	319.4	1932.7
CDKN2D	p19INK4d	2.67E-06	322.8	481.4
CCND1	CCND1	3.21E-24	8431.0	2324.0
CCNE1	CCNE1	7.67E-01	284.2	276.8
CDK2	CDK2	1.62E-26	892.8	1545.3
CDK4	CDK4	1.45E-03	3045.0	3582.9
CDK6	CDK6	5.20E-23	3148.5	958.2
TP53	p53	6.48E-20	1251.3	2773.7
MDM2	MDM2	3.97E-11	1751.7	2779.6

Table S2. E2F-Regulated Genes (ERG)

ACOX1
ACTA2
ADAMTS1
ANLN
ANXA8
APAF1
APBB2
APOE
AR
ARHGAP4
ASH2L
ATM
AXIN2
BACH1
BAD
BAK1
BARD1
BBC3
BCL2
BCL2L11
BCL3
BID
BIRC5
BLM
BMI1
BMP2
BMPR1A
BOK
BRCA1
BTG3
BUB1
BUB1B
BUB3
CALR
CASP3
CASP7
CASP8
CAV1
CBFB
CBX3

CBX5
CCNA2
CCNB1
CCNB2
CCND1
CCND3
CCNE1
CCNE2
CCNF
CCNG2
CD58
CDC20
CDC25A
CDC25B
CDC25C
CDC6
CDK2
CDKN1C
CDKN2C
CDKN3
CDS1
CENPA
CENPE
CENPF
CFLAR
CHAF1A
CHAF1B
CHEK1
CITED1
CITED2
CKS2
COL11A1
CPT1A
CSDA
CSRP2
CSTF1
CTGF
DCK
DDB2
DEK
DHFR

DHPS
DMRT1
DNMT1
DTYMK
DUT
DYRK1A
E2F1
E2F2
E2F3
ECT2
EED
EGR1
ENO3
EPS8
EZH2
FEN1
FGF2
FGFR2
FGFR3
FIGNL1
FOXM1
FST
GAB2
GADD45A
GADD45B
GCH1
GEM
H2AFX
H2AFZ
HEY1
HMMR
HOXA7
HOXA9
HRK
HUNK
ID3
IFNA2
INCENP
INHBA
INMT
ISYNA1

KIF4A
KPNA2
LHX2
LIG1
LMNB1
LSM5
MAD2L1
MAF
MAP2K1
MAP2K2
MAP3K14
MAP3K5
MAP3K7
MAPK1
MAPK3
MAPK4
MAPK8
MAPK9
MCL1
MCM2
MCM3
MCM4
MCM5
MCM6
MCM7
MFAP1
MKI67
MLH1
MMP16
MRE11A
MSH2
MTHFD1
MYB
MYC
NASP
NEK2
NFKB2
NOLC1
NPAT
NRP1
NUP107

NUP155
ORC2L
ORC3L
ORC6L
OSMR
PAWR
PBX3
PCNA
PDCD4
PDGFRA
PITX1
PLAU
PLSCR1
PMAIP1
PMF1
PMS2
PMS2L1
POLA2
POLD1
POLE2
PPM1D
PPP1R13B
PRC1
PRG4
PRIM1
PRKAR2B
PRPS1
PTTG1
RAD51
RAD51C
RAD52
RAD54L
RANBP9
RB1
RBL1
RECQL
RFC2
RFC3
RFC4
RFC5
ROD1

RPA1
RPA2
RPA3
RRM1
RRM2
SERPINE1
SERPINF2
SFPQ
SFRS2
SKP2
SLBP
SLPI
SMARCA2
SMARCA5
SNAPC1
SOX9
SPHK1
SRPR
STMN1
SULT2A1
TACC1
TANK
TCF19
TCOF1
TEAD4
TGFA
TGFB1I1
TGFB2
TGFB3
TIMELESS
TK1
TMPO
TNFSF9
TOP2A
TOPBP1
TP53BP2
TP73
TTK
TYMS
UMPS
UNG

USP1
VEGFB
VRK1
XRCC4
YY1
AURKB
FOXN3
KIF2C
CKS1B
PLK1
HIST1H2BE
GJC1
KIF20A
HIST2H4A
HMGB1
CDCA7L
HIST1H3D
DNA2
KIF22
HIST1H2AD
CDK1
FAS
PRIM2
CENPK
SRGAP2
SMC2
HSP90B1
UBE2C
HNRNPC
TPX2
CDCA4
RACGAP1
UHRF1
HIST1H2AE
SMC4
HIST1H2BJ
PBK
FURIN
PARP1
HIST1H2BN
CCNO

HMGB2
LIMA1
TBC1D2B
FOXO1
HIST1H2BO
HIST1H3B
HIST1H2BF
SHCBP1
UBE2T
HIST1H3E
KIF11
SIRPA
PEX19
CDC45
PLK4
GRAMD4
RAD51AP1
DBF4
SIVA1
MELK
HMGB3
FOXO3
ASF1B
FGFR1OP
HIST1H2AC
KIFC1
DIABLO
SSX2IP
KBTBD10
VIPR1
AURKA
NUSAP1
CDC7
GINS1
VEGFA
PLK2
FANCL
KIF20B
NDC80

Table S3. Uniquely Regulated Genes in HPV(-) Low ERG HNSC Tumors

SGOL1
KIF15
CCNA2
KIF11
CENPK
NUSAP1
RACGAP1
HMGB2
MSH2
RFC5
SMC4
RBMX
BUB1B
ZWINT
ASPM
HELLS
DEPDC1
CDCA7
BUB1
SKA1
PLK4
MCM6
CDC7
SGOL2
CENPF
NUF2
ZNF367
TOP2A
CIT
ERCC6L
YEATS4
CDCA2
BRCA1
TTK
CEP152
ARHGAP11A
ATAD5
PBK
RFC3
TRAIP

BRIP1
MCM3
MCM10
CEP78
CENPE
DBF4
ZWILCH
KIF20A
CDK1
CENPH
KIFC1
SKA3
XPO1
RMI1
WDR76
NEIL3
GEN1
C15orf42
PRIM1
DCK
KIF20B
GINS4
DEK
CHAF1B
CCDC18
HNRNPR
CDC25A
MSH6
DNA2
LRRCC1
SPC25
C12orf32
PRC1
MAD2L1
CCNB1
C14orf106
CDCA8
SMC2
POLE2
NCAPG2
CCDC21

KIF18A
SASS6
SFRS7
FANCI
NEK2
FBXO5
NCAPH
RRM2
ORC1L
LIN9
C14orf145
DNAJC9
POC1A
C21orf45
STIL
ASF1B
SNRNP40
NCAPG
SMC3
CKAP2L
RFC2
MND1
C17orf53
IQGAP3
LOC100128191
CPSF6
DCLRE1A
NLRX1
RRM1
CDC25C
MCM4
C4orf21
CCDC138
DNMT1
MKI67
DLGAP5
CCNB2
GINS2
TYMS
DHX9
MCM7

BLM
RCC1
DUT
UHRF1
KIF23
GSG2
POLQ
USP39
H2AFZ
CENPQ
NUP54
LYNX1
BRCA2
CDT1
C4orf46
CEP192
TOM1
C6orf167
HMMR
GINS1
SART3
ARHGAP11B
KHDRBS1
FANCM
CHAF1A
STMN1
KNTC1
WHSC1
CEP110
DEPDC1B
CENPW
METRNL
SCLT1
RAD54B
KIF4B
KIF18B
TROAP
CASP8AP2
HNRNPA1L2
PSRC1
GSTCD

HNRNPU
NUP107
C10orf119
TMEM97
POC5
ATP6V0D1
SPATA5
NCAPD3
CENPM
OIP5
HAT1
SNRNP48
KIAA1539
EPHB3
SMC6
TPX2
TOPBP1
TMEM79
TTF2
NCAPD2
EXO1
RBL1
ELAVL1
RAD51L3
FEN1
CHEK1
NDC80
C1orf112
XRCC2
ATAD2
KIF4A
CDC6
CENPI
CENPN
PHF19
ATP6V0A1
MELK
C11orf82
KRT6B
VRK1
LYPD3

TOLLIP
NIF3L1
FAM72B
SLC24A3
SRRT
SPAG5
KIAA0101
MTHFD1
IMMT
TAF5
ORC3L
KIF22
DCLRE1B
NASP
C13orf34
HNRNPL
C10orf137
TMEM194A
GTSE1
DSN1
CEP135
UBR7
NR2C2AP
CDK2
CASC5
GRN
H2AFV
POLE3
TFAM
RACGAP1P
PPIL5
SFRS2
C19orf40
EPR1
KRT6C
HJURP
METTL4
CLSPN
GMNN
S100A7
FANCC

EXOSC9
MPHOSPH9
RAD51AP1
DBF4B
PARP2
TIPIN
DTL
TIMELESS
SLC5A10
PNN
C12orf24
CST3
SPC24
PPP5C
RAD51
SF3A3
TMPRSS13
THEM5
HNRNPA2B1
TTF1
PARP1
WBP11
PRELID2
SENP1
POLD1
UBE2T
LIMK2
DKFZp779M0652
CCDC41
C2orf44
HNRNPD
SRRM1
FANCB
FAM72D
IVL
WDR67
MCM8
CTCFL
SULT2B1
CCNF
TRIM59

CCDC77
RBBP8
SKA2
UPF3B
HNRNPH3
CDC23
C9orf100
ADAP2
LSM6
FANCA
FANCG
CTDSPL2
KIAA0146
MASTL
MSH5
SNRPA
MVP
C12orf65
NUCKS1
WDHD1
DDX12
NAA40
MIS12
SSRP1
HMGB1
C21orf58
JUP
RNF11
LBR
ALG10
POLA2
C18orf56
AURKB
TMCO6
C13orf27
CDCA7L
KLK11
FOXM1
POLG2
KHSRP
KIAA1731

RPAP3
ARL6IP1
DMKN
UBA2
SFRS1
RAD54L
GLTPD1
POLR3K
DDX20
PCGF6
LOC642846
NARG2
ALMS1
PPP2R2C
HAUS6
PAFAH1B3
CHEK2
CENPL
CYB5R1
MCM3APAS
C2orf48
SNAPC5
TUBB2A
MRPL35
SLC22A23
TRIM29
SFN
BNIPL
IFT81
CDC20
CKS2
5-Sep
MUTYH
S100A12
AKAP1
MICALL1
NONO
SEPHS1
SNX33
C2orf3
PAXIP1

HMGXB4
TRIM37
IL1F5
DGKE
CHTF18
KRT10
GORAB
CENPA
RPA3
RECQL4
C19orf48
ECT2
C1orf174
KIF14
MAFB
KIAA1524
TFAP4
CCDC150
TMEM48
IGFL1
SNRNP25
SCN3B
FAHD1
SFPQ
KRT8
ILF2
ZRANB3
RBM12B
DFFB
ITPKA
ARL6IP6
NIPAL4
DCTPP1
HAUS8
SLC39A2
IFT80
LOC200030
KPNA2
WDR92
GAS2L3
MCM5

TRIP13
E2F7
CDC5L
PLEKHM1
TTC21B
POLR2D
CDA
ANKRD26
AMFR
CCDC45
CDC45
TOR3A
C7orf44
ORC6L
ZNF26
ZFYVE1
CCBP2
CCDC15
FAM64A
TREX2
MYO19
C18orf54
DHX35
SLC29A2
NUP85
ANP32E
KIF2C
MAPK3
ABCA12
SRBD1
TPR
TMEM185A
PLD2
SPRR2E
IMMP1L
NUDT1
PPP1CC
LRP10
ORC2L
KIF1C
POLD4

PDZK1IP1
SLC5A1
CCDC75
NUP160
RNASEH2A
TACC3
KLK6
CDK4
CSTF3
C15orf23
LIN52
NVL
LSM4
FAM54A
ANKRD36B
KCTD10
TRMT11
SLC15A1
PIF1
ZNF195
CCDC111
PLA2G4E
UIMC1
QTRTD1
ZNF530
SLC25A19
FAM72A
FBXO43
NUP62CL
OTUB2
AIM1L
RPIA
C1orf109
UBE2S
C14orf143
CEP57
LYPD5
C6orf132
C4orf3
RSPH3
RNASEH2B

MTBP
TUBGCP3
CTSA
DNAJB6
ITM2B
LGALS7B
NUP188
DARS2
NOD2
NCBP1
PROSC
CDCA3
VASN
NAP1L1
SMC1A
MCM2
GABPB1
TCERG1
PFKFB2
SKP2
GTF3C5
ALOX12B
C1orf135
TUBA1B
PPIH
NOL8
C9orf80
BIRC5
BAZ1B
MAK16
ARHGEF37
C6orf25
MDC1
CST6
PLCD1
SDR9C7
NUP35
PLIN3
TAF8
RNF39
LTB4R2

DLEU2L
DDX11
TAF6
CHML
TMEM86A
SET
EPB41L2
RAD21
NUP37
TFRC
ODF2
C10orf35
CENPO
PHF6
PTPLAD1
RCCD1
ACTL6A
TMEM40
CCDC148
PIM1
E2F5
CITED4
HNRNPK
CCDC163P
ZNF670
RHBDF1
RAVER1
TFDP1
MED8
MAF
LOC144438
AGPAT5
HYLS1
FIP1L1
LOC341056
DSCC1
ZBTB2
METTL10
ERCC8
PSMC3IP
RSRC2

S100A9
TPST2
GPR78
EME1
FAM174A
ATP6V0C
THOC1
RASAL1
NUP133
PRIM2
RFC4
AQP3
CEP55

Table S4. Genes Uniquely Upregulated in HPV(-) Low ERG HNSCC Tumors

KIAA1539
KRT6B
GRN
KRT6C
S100A7
SLC5A10
THEM5
LIMK2
ADAP2
MVP
JUP
DMKN
PPP2R2C
CYB5R1
TUBB2A
SLC22A23
SFN
S100A12
MICALL1
IL1F5
KRT10
MAFB
IGFL1
NIPAL4
CDA
TREX2
ABCA12
TMEM185A
SPRR2E
KLK6
SLC15A1
PLA2G4E
LYPD5
CTSA
LGALS7B
NOD2
ALOX12B
CST6
SDR9C7
LTB4R2

TMEM86A
CITED4
MED8
MAF
TPST2

Table S5. Differentially regulated networks in uniquely up-regulated genes in HPV(-) low ERG tumors.

ID	Molecules in Network	Score	Focus Molecules	Top Diseases and Functions
1	APOH,CRABP2,DSC2,DSG3,DUSP14,EHF,ERK,IgG,JUP,KLK5,KLK6,KLK7,KLK8,KLK11,KRT10,KRT14,KRT15,KRT16,KRT6B,KSR2,LGALS7/LGALS7B,LTB4R2,MVP,NFkB (complex),NOD2,PELI2,PPP2R2C,PPP2R5E,S100A7,S100A12,SPIB,SPRR2E,TAC4,TRIP6,TUBB2A	26	13	Dermatological Diseases and Conditions, Inflammatory Disease, Inflammatory Response
2	ABCA12,ALOX12B,AMOTL2,ATP1B3,CASP3,CD300C,CDA,E4F1,EHD1,GMPS,GRN,HIP1,IL36RN,LILRA5,LIMK2,LSS,MAF,MAFB,miR-17-3p (and other miRNAs w/seed CUGCAGU),miR-378a-3p (and other miRNAs w/seed CUGGACU),NINJ1,PPT1,RB1,SCO2,SEMA3C,SFN,SLC7A2,SQLE,SYVN1,TGM2,TINCR,TNF,TP53,UBQLN2,UGCG	16	9	Cell Death and Survival, Cellular Development, Cellular Compromise
3	MMP11,SDR9C7	2	1	Developmental Disorder, Cancer, Cardiovascular Disease
4	IGFL1,IGFLR1	2	1	Cancer, Gastrointestinal Disease, Organismal Injury and Abnormalities
5	CITED4,FGF8	2	1	Cardiovascular System Development and Function, Cellular Development, Cellular Growth and Proliferation
6	KRT6C,MGEA5	2	1	Cardiovascular System Development and Function, Dermatological Diseases and Conditions, Hereditary Disorder
7	DGKA,MICALL1,RAB13	2	1	Cell-To-Cell Signaling and Interaction, Cellular Assembly and Organization, Cellular Function and Maintenance
8	26s Proteasome,CTSA,GLB1	2	1	Developmental Disorder, Hereditary Disorder, Metabolic Disease
9	MED8,MED26,MED29,POLR2A,SPEN	2	1	Infectious Diseases, DNA Replication, Recombination, and Repair, Cancer

Table S6. Clinical correlations using Chi Squared Test

**p.value: 1.705203e-42** anatomic_organ_subdivision	HPV(-) high	HPV(-) low	HPV(+)
Alveolar Ridge	3	11	4
Base of tongue	5	6	16
Buccal Mucosa	11	8	0
Floor of mouth	25	32	1
Hard Palate	0	6	1
Hypopharynx	2	4	2
Larynx	51	56	2
Lip	1	2	0
Oral Cavity	22	42	5
Oral Tongue	33	84	4
Oropharynx	3	4	1
Tonsil	6	2	34

**p.value: 8.20524e-16** tumor_grade	HPV(-) high	HPV(-) low	HPV(+)
G1	10	48	1
G2	96	165	26
G3	49	41	28
G4	1	0	6
GX	5	3	7

**p.value: 1.786039e-07** clinical_N	HPV(-) high	HPV(-) low	HPV(+)
N0	80	134	18
N1	21	48	8
N2	6	8	4
N2a	3	5	9
N2b	24	26	23
N2c	18	20	4
N3	2	3	3
NX	8	9	1

**p.value: 0.0009039816**	HPV(-) high	HPV(-) low	HPV(+)
gender			
FEMALE	42	83	7
MALE	120	174	63

———————————————————————

**p.value: 0.0009852978**	HPV(-) high	HPV(-) low	HPV(+)
perineural_invasion			
NO	66	88	27
YES	54	99	6

———————————————————————

**p.value: 0.002194297**	HPV(-) high	HPV(-) low	HPV(+)
histologic_diagnosis			
Head and Neck Squamous (	160	255	65
Head and Neck Squamous (	0	1	0
Head and Neck Squamous (	2	1	5

———————————————————————

**p.value: 0.005728068**	HPV(-) high	HPV(-) low	HPV(+)
ajcc_tumor_pathologic_pt			
T0	0	1	0
T1	12	26	7
T2	41	64	23
T3	38	51	6
T4	4	4	1
T4a	53	84	10
T4b	1	3	0
TX	10	15	13

———————————————————————

**p.value: 0.007140524**	HPV(-) high	HPV(-) low	HPV(+)
ajcc_nodes_pathologic_pn			
N0	53	103	13
N1	26	26	8

N2	5	4	2
N2a	3	3	3
N2b	31	47	14
N2c	19	24	1
N3	4	2	1
NX	18	28	18

**p.value: 0.007787052**	HPV(-) high	HPV(-) low	HPV(+)
margin_status			
Close	20	20	6
Negative	115	188	27
Positive	16	27	13

**p.value: 0.00876613**	HPV(-) high	HPV(-) low	HPV(+)
vital_status			
Alive	102	168	58
Dead	60	89	12

**p.value: 0.008968685**	HPV(-) high	HPV(-) low	HPV(+)
ajcc_metastasis_pathologic_pm			
M0	54	94	19
MX	21	18	13

**p.value: 0.0100549**	HPV(-) high	HPV(-) low	HPV(+)
clinical_M			
M0	149	247	70
M1	2	1	0
MX	11	4	0

**p.value: 0.02056691**	HPV(-) high	HPV(-) low	HPV(+)
clinical_T			

T1	5	18	8
T2	46	67	30
T3	46	71	12
T4	6	12	3
T4a	50	82	14
T4b	2	0	1
TX	7	3	2

————————————————————————

**p.value: 0.02233873**	HPV(-) high	HPV(-) low	HPV(+)
tobacco_smoking_history_indicator			
Current reformed smoker	49	58	24
Current reformed smoker	15	45	9
Current Reformed Smoker	0	2	0
Current smoker	63	89	15
Lifelong Non-smoker	30	57	21

————————————————————————

**p.value: 0.02732582**	HPV(-) high	HPV(-) low	HPV(+)
alcohol_consumption_frequency			
0	13	28	9
1	5	10	5
2	4	5	1
2.5	0	0	1
3	3	1	2
4	6	1	3
5	2	2	2
6	4	0	0
7	35	54	11

————————————————————————

**p.value: 0.0379805**	HPV(-) high	HPV(-) low	HPV(+)
tumor_status			
TUMOR FREE	93	166	52
WITH TUMOR	54	66	13

————————————————————————

**p.value: 0.04655292**	HPV(-) high	HPV(-) low	HPV(+)

laterality

Left	47	62	22
Midline	11	19	1
Right	41	72	37

**p.value: 0.05163609**	HPV(-) high	HPV(-) low	HPV(+)
clinical_stage			
Stage I	3	14	2
Stage II	33	50	10
Stage III	33	61	7
Stage IVA	80	120	48
Stage IVB	4	3	3
Stage IVC	2	2	0

**p.value: 0.06669432**	HPV(-) high	HPV(-) low	HPV(+)
race			
AMERICAN INDIAN OR ALAS	0	2	0
ASIAN	1	10	0
BLACK OR AFRICAN AMERIC	16	16	3
WHITE	137	219	67

Table S7. Recurrent mutated genes in each HNSCC group that are not mutated in the other two groups.

HPV(-) low ERG		HPV(-) high ERG		HPV(+)	
**Gene**	**Count**	**Gene**	**Count**	**Gene**	**Count**
DCAF12L1	9	FRMPD2	7	CCBE1	3
DRP2	8	ZNF507	7	FCGR2A	3
ALCAM	7	FOXP1	6	ISY1	3
CASP8AP2	7	ATP11B	5	NAB2	3
KDM3B	7	ATP2A2	5	RAB2A	3
SEMA4G	7	GPR161	5	SLC25A19	3
SOX9	7	GYPA	5	SYT14	3
TNPO2	7	MFHAS1	5	ACP5	2
TUBD1	7	PLEKHH1	5	ANGPTL7	2
USP28	7	PSG2	5	AP1AR	2
ABCA5	6	SEC23B	5	ARSE	2
ATP2B4	6	THAP5	5	BIN3	2
CCDC7	6	ANP32E	4	C21orf128	2
GUCY2D	6	AQP9	4	CAPS	2
HTR6	6	CAPN10	4	CDSN	2
LINGO4	6	CC2D2B	4	CEP55	2
LONP2	6	CCL1	4	CINP	2
MAVS	6	CHIT1	4	COPZ2	2
NEK8	6	CLDN10	4	DPH3	2
OR10G8	6	CNNM3	4	EIF2S1	2
SGK2	6	COL10A1	4	EMX1	2
SH2D1A	6	CXXC4	4	FKBP11	2
STRN4	6	DDI1	4	FNDC7	2
USP6NL	6	DMRTC2	4	GGA3	2
A1BG	5	DPEP3	4	GNL1	2
ANKMY1	5	FAM171A1	4	HMG20B	2
AP3B2	5	FBXO30	4	HSPB8	2
ARMCX5	5	GPR115	4	KATNB1	2
BPI	5	HPSE	4	KIR3DL3	2
CA8	5	KRT6A	4	MAD2L2	2
CACNG6	5	LARP6	4	MED11	2
COCH	5	LPPR5	4	MYLPF	2
COL2A1	5	LZTFL1	4	NCSTN	2
CSHL1	5	MAPRE2	4	NRG4	2
CWC22	5	MCM3	4	NTNG2	2
CYP26B1	5	NFIB	4	OR1F1	2
DLG4	5	OR2A14	4	OTC	2
EFCAB4B	5	OR52A5	4	PCBP4	2
GLRA2	5	PAQR7	4	PHF7	2
GULP1	5	PRKAB1	4	POLR3D	2

HDAC2	5	PRSS48	4	PPP1CB	2
HIST1H4E	5	PSG5	4	PRKD2	2
IDUA	5	RAB37	4	PSPN	2
IKBKE	5	RIMS3	4	RIOK1	2
KCTD1	5	RRAGC	4	RNF113A	2
KPNA6	5	SGK3	4	SLC3A1	2
LILRB3	5	SH3BP2	4	SMARCA5	2
MDH1B	5	SLC25A6	4	SPRR2E	2
MEPE	5	SPRED2	4	SUV39H2	2
MLLT6	5	TAB2	4	TBX10	2
MYO6	5	TDO2	4	TOLLIP	2
NEUROD1	5	TMBIM4	4	TOP1	2
NKAP	5	UNC50	4	TRAIP	2
PPM1D	5	USP44	4	TRIM59	2
RAB3IP	5	VEGFC	4	UBQLN1	2
RAD51AP1	5	ZBTB40	4	ZER1	2
SCIN	5	ZCCHC7	4	ZNF627	2
SLC22A2	5	ZNF442	4		
SLC5A1	5	ZNF641	4		
STAG1	5	ZNF83	4		
TRIM43	5	AATF	3		
USP5	5	ABHD5	3		
ZBTB3	5	ACBD4	3		
ZDHHC8	5	ACSF3	3		
ZNF271	5	ADCK2	3		
ACAP2	4	ALG11	3		
ACRBP	4	ALPP	3		
ACTRT2	4	ANKRD24	3		
ADORA1	4	ARHGAP18	3		
ALOX5	4	ARHGEF9	3		
ALPL	4	ASB10	3		
AOC2	4	ATP6V1B1	3		
APOL4	4	AURKB	3		
ARHGEF11	4	B3GNT1	3		
ATXN7	4	BDKRB2	3		
C6orf70	4	BEND7	3		
CDC42	4	BRAP	3		
CEACAM3	4	BTG3	3		
CEP63	4	C17orf104	3		
CPN1	4	C1QTNF7	3		
CPT1B	4	C6orf10	3		
CTU2	4	C6orf106	3		
DFNA5	4	CABP4	3		

DNAJB7	4	CACNG3	3
DSPP	4	CADM4	3
DTNB	4	CCDC126	3
DUSP26	4	CCDC19	3
EIF3G	4	CCDC28B	3
FAM27L	4	CDC14A	3
FPR2	4	CDH15	3
GLB1	4	CDR2	3
GPR39	4	CMKLR1	3
GRIN3B	4	CPA4	3
HK1	4	CRTAC1	3
HKR1	4	CTH	3
HMGCLL1	4	CYP27B1	3
ITGB2	4	DCST2	3
KCNAB1	4	DDX18	3
KCNJ8	4	DDX23	3
KCNK9	4	DES	3
KIF9	4	DYRK2	3
LGI4	4	ENO3	3
LGSN	4	EPHB2	3
LPXN	4	FAIM3	3
MAPK10	4	FAM13B	3
MED30	4	FAM171B	3
MYLK3	4	FKBP4	3
NBN	4	FUT3	3
NCDN	4	GPT2	3
NEDD4L	4	HHLA3	3
NEO1	4	HIST1H2BN	3
OR14J1	4	HSDL1	3
OR1A2	4	IL5RA	3
OR52D1	4	INF2	3
OR52I2	4	IQCB1	3
OR52L1	4	KCTD4	3
OR6C70	4	KLK13	3
PAPOLB	4	KRT6C	3
PHLPP2	4	LGALS9B	3
PRPF38B	4	LHFP	3
PSD2	4	LRMP	3
PTX3	4	LRRFIP2	3
RNF128	4	MAPK8IP2	3
SEC24D	4	MEIS3	3
SENP1	4	MSN	3
SHC4	4	MTL5	3

SLAIN2	4	NBPF15	3
SLC6A13	4	NCR2	3
SLC6A17	4	NFIC	3
SMAD3	4	NPR1	3
SMG5	4	NXF5	3
SOX2	4	OGFR	3
SPHK2	4	OLFML1	3
SYT5	4	OR1L8	3
SYT9	4	OSBPL11	3
TCP10L2	4	P2RY8	3
TEX9	4	PAPLN	3
TLE6	4	PBLD	3
TMEM206	4	PDLIM3	3
TPI1	4	PIGN	3
TRIM33	4	PPIG	3
TTC23L	4	PPP1R3D	3
UBXN6	4	PRRG1	3
VPS16	4	PTDSS2	3
WNT3	4	PYHIN1	3
ZFP36	4	RAB25	3
ZNF443	4	RAPH1	3
ZNF800	4	REN	3
AARS	3	RHAG	3
ABHD15	3	RING1	3
ABO	3	RIT2	3
ACOT8	3	RTN4RL1	3
ADAM15	3	SERPINB8	3
ADAMTS1	3	SIGLEC5	3
AGPAT3	3	SIK2	3
ALDH3A2	3	SLC36A4	3
AMACR	3	SMC1A	3
ANGPTL3	3	SMYD5	3
AP4M1	3	SRFBP1	3
APCDD1	3	STXBP6	3
APOL1	3	TAS1R2	3
ARHGEF2	3	TBL1Y	3
ARMC10	3	TGFBRAP1	3
ASB18	3	TINAG	3
ASTL	3	TLR2	3
ATF7	3	TMEM154	3
ATP5G2	3	TOR1AIP1	3
ATP9B	3	TRAF3IP2	3
AVEN	3	TRIT1	3

B3GALTL	3
BCAS2	3
C15orf32	3
C19orf18	3
C1orf63	3
C3orf37	3
C4orf50	3
C6orf62	3
C9orf117	3
CARKD	3
CCBL1	3
CCDC105	3
CCDC74A	3
CCL25	3
CDC45	3
COQ10A	3
CPEB4	3
CSF3R	3
CSPG5	3
CTDSPL2	3
CTNNBL1	3
CUZD1	3
DCDC2	3
DDHD2	3
DHTKD1	3
DMPK	3
DNAJA3	3
DYRK3	3
EFNB1	3
EIF5	3
EMR3	3
ESRP2	3
ETNK2	3
ETS1	3
EXD1	3
EXT1	3
EXTL2	3
FAM134A	3
FAR2	3
FBXL19	3
FBXL20	3
FBXL5	3
FCN2	3
UBE3C	3
UNK	3
USP30	3
VRK2	3
VWA2	3
WDR45	3
WDR76	3
WDR92	3
WDYHV1	3
ZBED1	3
ZBTB7A	3
ZNF436	3
ZNF566	3
ZNF81	3
ZNF823	3

FEZ1	3
FOXO1	3
GAB3	3
GANAB	3
GAS8	3
GBA	3
GFI1B	3
GIN1	3
GLTPD1	3
GMPR2	3
HBS1L	3
HDAC11	3
HIST1H2AK	3
HNRNPA1L2	3
HORMAD1	3
HS3ST6	3
IFI44	3
IGFBP1	3
IL27RA	3
IQCA1	3
ITGB1BP2	3
ITPK1	3
KBTBD6	3
KLHDC8B	3
KLHL2	3
KPNA3	3
KRBA1	3
KRT9	3
LDHC	3
LEMD3	3
LRRC14	3
LRRC42	3
LRRIQ4	3
MAGEB2	3
MAPK8IP1	3
MATK	3
MEA1	3
MEF2A	3
MORC4	3
MOXD1	3
MPHOSPH9	3
MRO	3
MST4	3

MTERFD1	3
MUM1L1	3
MUTYH	3
NAB1	3
NCBP2	3
NCF4	3
NCKIPSD	3
NECAB2	3
NFE2	3
NHLRC3	3
NOSIP	3
NPVF	3
NR2F1	3
NT5DC1	3
NUDT19	3
OR10H2	3
OR11A1	3
OR13A1	3
OR1J2	3
OR4X2	3
OR52R1	3
OR6C74	3
OSBPL2	3
OSR1	3
PACRGL	3
PAPD5	3
PARK2	3
PARP15	3
PDGFC	3
PDPR	3
PEX14	3
PEX3	3
PHEX	3
PHLDA2	3
PHYHD1	3
POLH	3
POLR1C	3
POU3F4	3
POU6F1	3
PPP1R3B	3
PRSS54	3
PSG1	3
PSG4	3

PUS7	3
RASSF2	3
RFC1	3
RGS1	3
RNF175	3
RNF214	3
RPS6KA4	3
S1PR1	3
S1PR3	3
SAMD11	3
SCAPER	3
SCARA3	3
SCML1	3
SDCBP	3
SEC61A2	3
SESN3	3
SFTPA1	3
SGCB	3
SIAH2	3
SKIL	3
SLC15A4	3
SLC25A12	3
SLC26A4	3
SLC6A16	3
SLC6A4	3
SMPDL3B	3
SPAM1	3
SPDYE3	3
SPIB	3
SPIN3	3
SPRED1	3
SRPR	3
SRPX2	3
SSRP1	3
STK38	3
SULT1C2	3
SYCE1	3
TADA2B	3
TBC1D5	3
TC2N	3
TCTN3	3
TEKT1	3
TFPT	3

TGFB1I1	3
TGFB2	3
THEM5	3
TIGD3	3
TIMM50	3
TMED5	3
TMEM106B	3
TMEM52	3
TNFRSF10A	3
TNFRSF11A	3
TNNI3K	3
TOR1AIP2	3
TRIM22	3
TSHR	3
TSPAN19	3
TTC22	3
TTLL8	3
TUB	3
TUBGCP5	3
TUSC5	3
UCP2	3
VWC2	3
WDR83	3
WNT8B	3
XAGE5	3
XPO6	3
ZBTB17	3
ZBTB32	3
ZBTB45	3
ZDHHC16	3
ZIC5	3
ZNF185	3
ZNF212	3
ZNF25	3
ZNF433	3
ZNF514	3
ZNF574	3
ZNF7	3
ZNHIT6	3

Table S8. Chi-squared test of mutational correlation across ERG-defined tumor groups

**Gene**	**p-value**
TP53	1.19E-41
CYLD	2.61E-07
FGFR3	7.42E-07
ZNF750	3.98E-06
CASZ1	3.99E-06
CDKN2A	4.59E-06
LRFN5	1.65E-05
FAT1	2.17E-05
KIAA1407	3.68E-05
GCKR	9.49E-05
HRC	9.49E-05
CCBE1	0.00010018
FCGR2A	0.00010018
ISY1	0.00010018
NAB2	0.00010018
RAB2A	0.00010018
SLC25A19	0.00010018
SYT14	0.00010018
PDE7A	0.00010693
PLK3	0.00010693
DCC	0.00014158
POLR3A	0.00037615
PRPF6	0.00037615
USH2A	0.00047616
UBE3A	0.0005094
FRMPD2	0.00065993
ZNF507	0.00065993
IMMT	0.00075988
WASF2	0.00075988
FAM47C	0.00085141
CCDC136	0.00094384
STAT3	0.00094384
PRDM9	0.00100261
ASAP1	0.00127103
B2M	0.00136985
IGF2R	0.00136985
C19orf57	0.00162153
CDC40	0.00162153
IFNGR1	0.00162153

IGSF9	0.00162153
SPATA6	0.00162153
UBOX5	0.00162153
ZCCHC2	0.00162153
ASPSCR1	0.00188274
CNR1	0.00188274
FLOT2	0.00188274
MAFB	0.00188274
MFSD2A	0.00188274
PVRL2	0.00188274
RUFY1	0.00188274
SEC24C	0.00188274
ZNF543	0.00188274
FOXP1	0.00190323
ACP5	0.00218431
ANGPTL7	0.00218431
AP1AR	0.00218431
ARSE	0.00218431
BIN3	0.00218431
C21orf128	0.00218431
CAPS	0.00218431
CDSN	0.00218431
CEP55	0.00218431
CINP	0.00218431
COPZ2	0.00218431
DPH3	0.00218431
EIF2S1	0.00218431
EMX1	0.00218431
FKBP11	0.00218431
FNDC7	0.00218431
GGA3	0.00218431
GNL1	0.00218431
HMG20B	0.00218431
HSPB8	0.00218431
KATNB1	0.00218431
KIR3DL3	0.00218431
MAD2L2	0.00218431
MED11	0.00218431
MYLPF	0.00218431
NCSTN	0.00218431
NRG4	0.00218431

NTNG2	0.00218431
OR1F1	0.00218431
OTC	0.00218431
PCBP4	0.00218431
PHF7	0.00218431
POLR3D	0.00218431
PPP1CB	0.00218431
PRKD2	0.00218431
PSPN	0.00218431
RIOK1	0.00218431
RNF113A	0.00218431
SLC3A1	0.00218431
SMARCA5	0.00218431
SPRR2E	0.00218431
SUV39H2	0.00218431
TBX10	0.00218431
TOLLIP	0.00218431
TOP1	0.00218431
TRAIP	0.00218431
TRIM59	0.00218431
UBQLN1	0.00218431
ZER1	0.00218431
ZNF627	0.00218431
KCNV1	0.00226475
LRRC37B	0.00236998
RGPD3	0.00241233
ZNF737	0.0029366
TMCC1	0.00314486
TMC5	0.00332407
PDZD2	0.00403455
NPTX2	0.00430089
SDK2	0.00430089
NLRC5	0.00431669
DDX60	0.00507565
SIN3B	0.00507565
ATP11B	0.00546491
ATP2A2	0.00546491
GPR161	0.00546491
GYPA	0.00546491
MFHAS1	0.00546491
PLEKHH1	0.00546491

PSG2	0.00546491
SEC23B	0.00546491
THAP5	0.00546491
STK11	0.00568997
UBE2Q1	0.00568997
SEMA3E	0.00570458
EPHA7	0.00595071
SPON1	0.00633617
DDX3X	0.00658173
KIAA1755	0.00658173
ZNF211	0.00658173
SLC35F4	0.00706217
CASP8	0.00716753
PCDHB8	0.00744123
KDM2B	0.00910389
AAK1	0.00918558
FAS	0.00918558
GNA15	0.00918558
KRT38	0.00918558
MED29	0.00918558
MVP	0.00918558
XIRP2	0.00918915
C17orf70	0.0092765
LIMK2	0.0092765
NPAS4	0.0092765
ANKRD30A	0.00944802
MYH13	0.00962007

Table S9. Mutational correlation of Rb-E2F/p53 pathway genes in HPV(+) and HPV(-) tumors

RB1 pval=0.02307788

	**0**	**1**
**-**	428	12
**+**	62	6

RBL1 pval=0.1378658

	**0**	**1**
**-**	433	7
**+**	65	3

RBL2 pval=0.5797222

	**0**	**1**
**-**	435	5
**+**	67	1

E2F1

	**0**
**-**	440
**+**	68

E2F2 pval=1

	**0**	**1**
**-**	437	3
**+**	68	0

E2F3 pval=0.350814

	**0**	**1**
**-**	438	2
**+**	67	1

E2F4 pval=1

	**0**	**1**
**-**	438	2

+	68	0

E2F5 pval=0.5797222

	0	1
-	435	5
+	67	1

E2F6 pval=0.1338583

	0	1
-	440	0
+	67	1

E2F7 pval=0.08856862

	0	1
-	438	2
+	66	2

E2F8 pval=0.4382292

	0	1
-	437	3
+	67	1

CDKN1A

	0
-	440
+	68

CDKN1B pval=0.350814

	0	1
-	438	2
+	67	1

CDKN1C

	0

-	440
+	68

CDKN2A pval=1.804049e-08

	0	1
-	327	113
+	68	0

CDKN2B

	0
-	440
+	68

CDKN2C pval=1

	0	1
-	439	1
+	68	0

CDKN2D

	0
-	440
+	68

CCND1 pval=1

	0	1
-	436	4
+	68	0

CCNE1 pval=0.5140237

	0	1
-	436	4
+	67	1

CDK2

	0
-	440
+	68

CDK4 pval=1

	0	1
-	435	5
+	68	0

CDK6 pval=1

	0	1
-	438	2
+	68	0

TP53 pval=1.190944e-40

	0	1
-	81	359
+	67	1

MDM2 pval=1

	0	1
-	438	2
+	68	0

Table S10a

Normalized Count RNA Prediction Analysis (Confusion Matrix)

		Reference		
		HPV(-) High ERG	HPV(-) Low ERG	HPV(+)
**Prediction**	HPV(-) High ERG	58	75	20
	HPV(-) Low ERG	58	111	17
	HPV(+)	48	77	35

Normalized Count RNA Prediction Statistics

	HPV(-) High ERG	HPV(-) Low ERG	HPV(+)
Sensitivity	0.3537	0.4221	0.48611
Specificity	0.7164	0.6822	0.70726
Pos Pred Value	0.3791	0.5968	0.21875
Neg Pred Value	0.6936	0.5144	0.89086
Prevalence	0.3287	0.5271	0.14429
Detection Rate	0.1162	0.2224	0.07014
Detection Prevalence	0.3066	0.3727	0.32064
Balanced Accuracy	0.535	0.5521	0.59669

Table S10b

123 Categorized RNA Prediction Analysis (Confusion Matrix)

		Reference		
		HPV(-) High ERG	HPV(-) Low ERG	HPV(+)
**Prediction**	HPV(-) High ERG	141	28	3
	HPV(-) Low ERG	20	234	2
	HPV(+)	3	1	67

123 Categorized RNA Prediction Statistics

	HPV(-) High ERG	HPV(-) Low ERG	HPV(+)
Sensitivity	0.8598	0.8897	0.9306
Specificity	0.9075	0.9068	0.9906
Pos Pred Value	0.8198	0.9141	0.9437
Neg Pred Value	0.9297	0.8801	0.9883
Prevalence	0.3287	0.5271	0.1443
Detection Rate	0.2826	0.4689	0.1343
Detection Prevalence	0.3447	0.513	0.1423
Balanced Accuracy	0.8836	0.8983	0.9606
