# Supplementary figures and images for: Identification of Head and Neck Cancer Subtypes Based on Human Papillomavirus Presence and E2F-Regulated Gene Expression
Source: mSphere. 2018 Jan 10;3(1):e00580-17. doi: 10.1128/mSphere.00580-17 (PMC5760753; doi:10.1128/mSphere.00580-17)

Figure S1

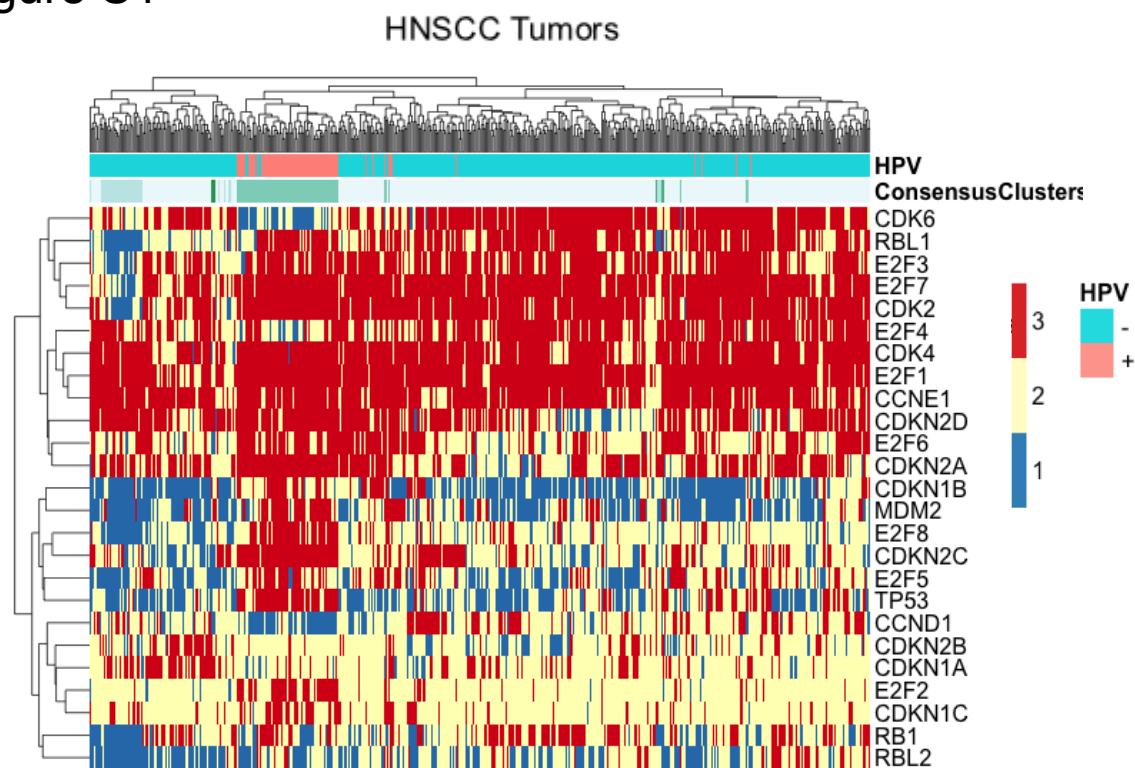

Supplement: FIG S1 [file sph001182443sf1.pdf]

Figure S2

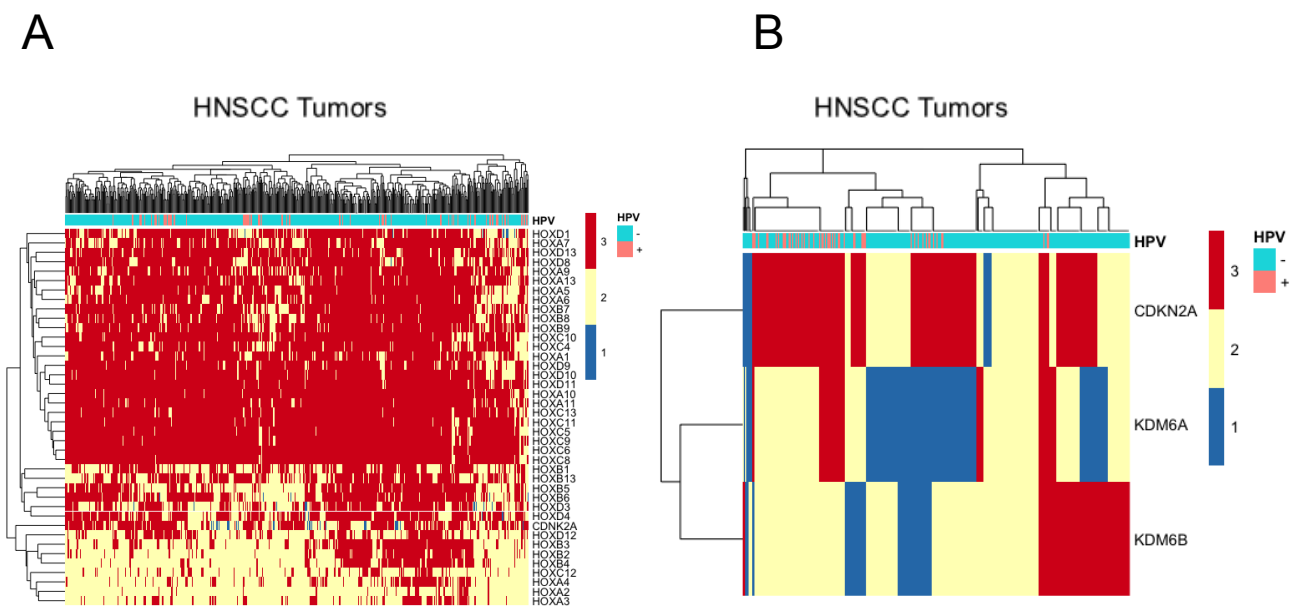

Supplement: FIG S2 [file sph001182443sf2.pdf]
