# Supplementary material for: Identification of Head and Neck Cancer Subtypes Based on Human Papillomavirus Presence and E2F-Regulated Gene Expression
Source: mSphere. 2018 Jan 10;3(1):e00580-17. doi: 10.1128/mSphere.00580-17 (PMC5760753; doi:10.1128/mSphere.00580-17)
Supplement: TEXT S1 [file sph001182443s1.pdf]

Supplementary Methods

Mutational Signature Analysis

The three ERG-defined tumor groups were analyzed to identify possible mutational signatures. First, a genome-wide search was run to look for genes that were mutated in one group, but not in the other two groups (**Table S7**). Genes are reported that were found to be mutated in three or more samples in the HPV(-) low ERG and HPV(-) high ERG groups, and two or more samples in the HPV(+) group (due to the low number of HPV(+) samples). While there were many genes reported, the count of mutations was not highly significant due to the relatively low number of samples. An addition, we examined mutational signatures using a chi-square analysis looking for significant differences in the number of mutations in each gene in relation to the ERG-defined groups (**Table S8**). TP53 was found to be the most significantly mutated gene across the three groups, but the two HPV(-) groups of tumors had similar representation of TP53 mutations. Thus, the analysis did not identify any significant differences between HPV(-) low ERG and HPV(-) high ERG tumors. An analysis of possible mutational signatures with genes curated from the COSMIC Cancer Gene Census also gave no significant results (data not shown).

Analysis of Categorization Method

An ANOVA test was used to identify differentially regulated genes between the three ERG-classified tumor groups. This type of analysis was run twice on two different sets of data: the normalized count dataset and the categorized dataset. In each experiment, RNA levels were compared across all three ERG tumor groups for every gene. Genes with a p-value < 1e-30 were selected, which identified 467 differentially regulated genes in the normalized count data, and 208 differentially regulated genes in the categorized data. In each dataset, the differentially regulated genes were run through a prediction analysis (R package 'MASS'), which uses linear discriminant analysis (LDA) to separate the tumors into classes based on a linear combination of features. Thus, tumors were sorted into the three ERG groups based on their distinct regulation of these genes. After categorizing these tumors through a prediction analysis, the R caret package was used to compare these models to the reference groups (the three ERG-based subgroups). This analysis was conducted for both the original normalized count RNA values and the categorized mRNA levels in order to compare the ability of the data to be grouped effectively. The results show that the normalized count RNA assemble together less accurately (**Supplementary Table 10a**) than the categorized data (**Supplementary Table 10b**). For example, only 58/164 high ERG HPV(-) tumors were predicted to fall into this category based on the normalized count analysis (caret results: accuracy of 0.4088, p-value 2.995e-11), while 141/164 of the high ERG HPV(-) tumors were predicted to fall into this category from the categorized analysis (caret results: accuracy of 0.8858, p-value < 2e-16). A stronger prediction analysis will have a higher number of tumors fall into the same reference group as the prediction group, which is seen in the categorized analysis. Thus, the categorization method seems to be a more accurate method for clustering tumors when using RNA expression levels.

Cutoff Determination for Categorization

In certain cases, the categorization of RNA levels may be used with a cutoff value to discard any genes with insignificant RNA levels in both the normal tissue and tumors. When a cutoff is applied, genes will be classified via the categorization method as previously described when their RNA levels are greater than the specified cutoff value in both the normal tissue and tumor. However, if the gene's RNA levels are lower than the cutoff in normal tissue, but higher than the cutoff in the tumor, it will be classified as up-regulated and given a value of 3. Similarly, if the RNA levels are higher than the cutoff in normal tissue, but below the cutoff in the tumor, the gene will be classified as down-regulated and given a value of 1. If the RNA levels in both tissues are below the cutoff, the gene will be classified as not applicable (NA) and removed from the data analysis. In **Figure 2B**, an expression cutoff of 1 was used for categorization across 499 tumors which resulted in the removal of 3,640 genes. The median number of tumors that contained insufficient expression for these genes was 470 with a minimum of 10 and a maximum of 499, which supports the removal of these genes. The categorization of Rb-E2F/p53 pathways genes was analyzed both with and without the cutoff, and the differences between the two analyses were insignificant (data not shown).
